# The Association between Ultra‐Processed Foods and Depression, Anxiety and Sleep in Adults: A Cross‐Sectional Study in Iran

**DOI:** 10.1002/fsn3.70316

**Published:** 2025-07-20

**Authors:** Niloufar Abdollahpour, Seyedeh Atieh Mousavi Fard, Alae Salahmanesh, Hossein Hatamzadeh, Reza Moeini, Sara Saffar Soflaei, Najmeh Seifi, Majid Ghayour‐Mobarhan

**Affiliations:** ^1^ Department of Nutrition, Faculty of Medicine Mashhad University of Medical Sciences Mashhad Iran; ^2^ Student Research Committee Mashhad University of Medical Sciences Mashhad Iran; ^3^ Metabolic Syndrome Research Center Mashhad University of Medical Sciences Mashhad Iran

**Keywords:** anxiety, depression, mental health, sleep, ultra‐processed foods

## Abstract

The increasing consumption of ultra‐processed foods (UPFs) has raised concerns about their impact on human health, yet their relationship with mental health remains debated. This study aimed to investigate the association between UPF intake and sleep adequacy, as well as the severity of anxiety and depression in Iranian adults. A cross‐sectional analysis was conducted on 5476 adults aged 35–65 years from the MASHAD study. Dietary intake was assessed using a validated food frequency questionnaire, and UPFs were classified based on the NOVA system. Depression and anxiety severity were measured using the Beck Depression Inventory (BDI) and Beck Anxiety Inventory (BAI), respectively. Sleep adequacy was defined as more than seven 7 h of sleep per night, based on self‐reported data. Higher UPF consumption was linked to increased odds of severe depression (OR = 1.25, 95% CI: 1.01–1.54), but this association lost statistical significance after full adjustment (OR = 1.25, 95% CI: 0.99–1.57). Subgroup analysis revealed a significant association in females, where high UPF intake was associated with a 44% higher risk of severe depression (OR = 1.44, 95% CI: 1.09–1.90, *p* = 0.01), whereas no such link was observed in males (*p* > 0.05). No significant associations were found between UPF intake and anxiety severity or sleep adequacy in either the overall population or gender subgroups (all *p* > 0.05). These findings suggest a possible association between UPF intake and depression severity among women, but not with anxiety or sleep. Further prospective and experimental studies are recommended to explore these relationships.

## Graphical abstract

This cross‐sectional study examined the link between ultra‐processed food (UPF) consumption and mental health indicators in 5476 Iranian adults. Higher UPF intake was associated with increased odds of severe depression, particularly in females, while no significant associations were found for anxiety or sleep adequacy. These findings highlight the potential impact of diet on mental well‐being, warranting further research into underlying mechanisms.

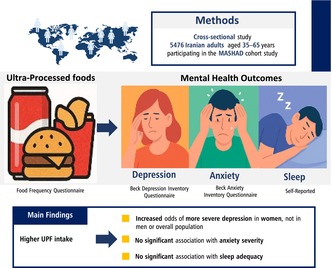

## 1 Introduction

Mental disorder is a well‐established and a major public health concern (Cabanas‐Sánchez et al. 2022; Zhang et al. 2021). On a global scale, it is estimated that depression affects 28.18% of individuals, anxiety impacts 29.57%, and insomnia influences 23.50% of adults aged 18 years and older based on 2023 data (Mahmud et al. 2023). In Iran, approximately 8.59% of adults aged 45–60 experience mental health disorders, with depression and anxiety being the most prevalent (Talebi et al. 2024). Mental disorders are among the leading contributors to the global instances of disease (Santomauro et al. 2021). Depression ranks as the second most important reason behind years lived with disability (YLDs), while anxiety ranks ninth (Risal et al. 2016). These disorders not only impair individual well‐being but also impose substantial financial and social burdens on healthcare systems worldwide (Santomauro et al. 2021). They are also associated with a rising risk of chronic illnesses, reduced quality of life, and exacerbation of sleep problems (Fang et al. 2019; Vadakkiniath 2023). In turn, sleep disturbances further intensify psychological symptoms and jeopardize overall health (Vestergaard et al. 2024). Antidepressant medications and brief psychotherapeutic interventions such as interpersonal psychotherapy, cognitive‐behavioral therapy, and problem‐solving therapy are commonly employed in the treatment of mental health conditions (Kohn et al. 2018). However, these therapies are often limited in accessibility and often have low adherence rates (Post 2015). Therefore, managing mental health disorders through lifestyle modifications may present an appealing option for healthcare providers.

In this context, dietary patterns, particularly the intake of ultra‐processed foods (UPFs), have emerged as a modifiable risk factor. Over recent decades, the global consumption of UPFs has risen dramatically. These foods, predominantly consumed in wealthy countries, have also experienced rapid growth in popularity in middle‐income nations (Monteiro et al. 2013). The NOVA classification system classifies foods into four categories based on their level of processing, placing UPFs in the most highly processed group (Fardet and Rock 2019). UPFs include items such as reconstituted meat products, chips, sugar‐sweetened beverages, cookies, breakfast cereals, packaged snacks, and various other convenience foods (Robinson and Johnstone 2024). These products typically have low nutritional value due to extensive processing, which increases their content of added sugars, saturated fats, and salt, while significantly reducing their levels of protein, fiber, vitamins, and minerals (Hecht et al. 2022). Given the biological plausibility of diet‐mental health interactions, such as via inflammation, gut microbiota imbalance, or nutrient deficiencies, UPFs may be linked to increased risks of psychological distress (Foster and Neufeld 2013; Sánchez‐Villegas et al. 2015; Sanchez‐Villegas et al. 2018).

In addition to these mechanisms, there is increasing evidence that sex differences may influence the relationship between dietary factors and mental health outcomes. Biological elements, such as hormonal fluctuations (e.g., variations in estrogen and progesterone levels), impact neurotransmission and stress responses differently in men and women (Wardle et al. 2004; Zheng et al. 2020). Psychosocial factors, including gender‐specific stressors and coping styles, also contribute to these differences. Recent systematic reviews have indicated that women may be more vulnerable than men to the negative mental health effects of poor diet quality and greater UPF consumption (Solomou et al. 2023; Xiong et al. 2024), emphasizing the need for sex‐specific analyses in diet and mental health research.

Despite growing interest in this area, recent systematic reviews and meta‐analyses suggest a possible positive association between UPF intake and depression (Barbaresko et al. 2024; Lane et al. 2024) although findings for anxiety remain inconclusive (Mazloomi et al. 2023; Lane et al. 2022). Additionally, these reviews are primarily based on studies from Western populations. Given the multifactorial etiology of depression and anxiety, which includes environmental, genetic, and nutrigenomic factors, the findings may be limited in their generalizability to other ethnic or regional groups (Ghernati et al. 2025). In the context of Iran, limited studies have examined UPF intake and mental health, and they often focus on specific groups such as women (Hosseininasab et al. 2024), students (Bazyar et al. 2020), or particular occupation groups (Janmohammadi et al. 2021). While some of these few investigations have reported negative associations with quality of life or mental health (Hosseininasab et al. 2024; Bazyar et al. 2020; Janmohammadi et al. 2021), the generalizability to the broader population remains unclear. To address these gaps, this study examined the relationship between UPF consumption and mental health outcomes in the Mashhad Stroke and Heart Atherosclerotic Disorder (MASHAD) population. In particular, alongside the assessment of depression and anxiety, sleep adequacy was evaluated as a key indicator of mental health. Additionally, given the possible influence of gender differences on these associations, analyses were performed separately for men and women. It was hypothesized that higher UPF intake would be associated with more severe depression and anxiety and a higher likelihood of inadequate sleep.

## 2 Methods

### 2.1 Study Population and Design

**FIGURE 1:**
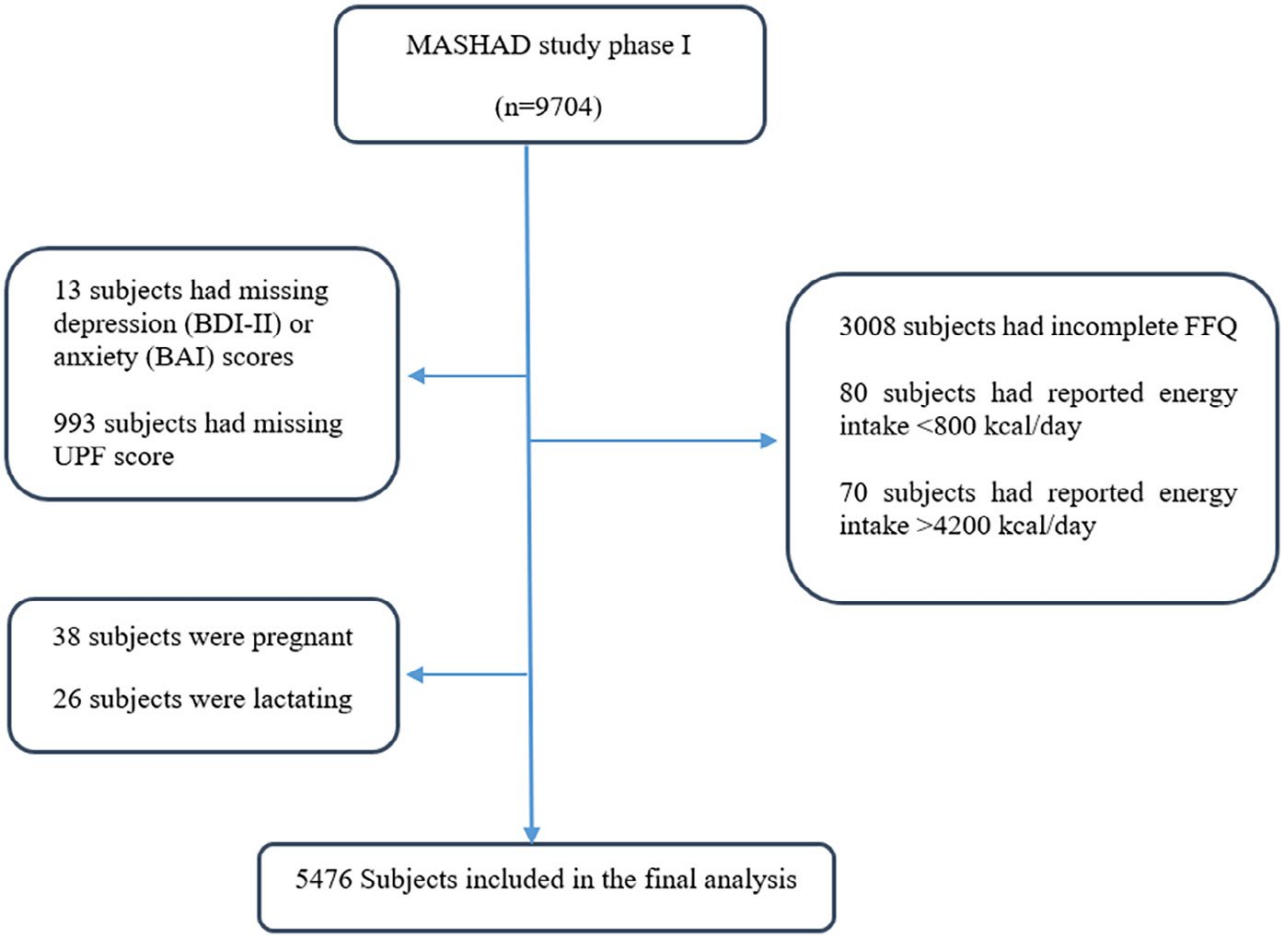
Flow diagram of study subject subgroups analyses. BAI: Beck's anxiety inventory; BDI‐II: Beck depression inventory‐II; FFQ: food frequency questionnaire; UPF: ultra‐processed food.

A total of 5476 participants from the MASHAD study were included in our cross‐sectional study. This study is a secondary analysis drawing on the baseline data of the MASHAD study, a cohort study spanning a decade, beginning in 2010 in northeastern Iran and including 9704 participants aged 35–65. It employs a stratified cluster‐randomized design, with each region in Mashhad divided into nine areas based on the divisions of the Mashhad Healthcare Center. More details have been provided in a previous study (Ghayour‐Mobarhan et al. 2015). In this study, individuals with incomplete data on anxiety or depression scores (*n* = 13), missing UPF score (*n* = 993), incomplete FFQ data (*n* = 3008), reported energy intake < 800 kcal/day (*n* = 80) or > 4200 kcal/day (*n* = 70), as well as those who were pregnant (*n* = 38) or lactating (*n* = 26), were excluded. The final participants analyzed included 5476 individuals (Figure 1). The sample size was based on the availability and completeness of data within the MASHAD cohort study. The large number of participants is considered sufficient to ensure adequate statistical power for the analyses conducted. This study was approved in accordance with the ethical guidelines of the Helsinki Declaration and Mashhad University of Medical Sciences (IR.MUMS.MEDICAL.REC.1403.412). Informed consent was obtained from all participants.

### 2.2 Anxiety and Depression Assessment

All participants completed the reliable and validated Persian versions of depression and anxiety questionnaires, administered by a healthcare professional (Ghassemzadeh et al. 2005; Kaviani and Mousavi 2008). Beck's Anxiety Inventory (BAI) was utilized to determine anxiety levels. The Persian version of the BAI has been previously validated, with Cronbach's alpha coefficient of 0.92 (Kaviani and Mousavi 2008). Anxiety levels were classified as minimal or mild (0–21), moderate (Xiong et al. 2024; Barbaresko et al. 2024; Lane et al. 2024; Mazloomi et al. 2023; Lane et al. 2022; Ghernati et al. 2025; Hosseininasab et al. 2024; Bazyar et al. 2020; Janmohammadi et al. 2021; Ghayour‐Mobarhan et al. 2015; Ghassemzadeh et al. 2005; Kaviani and Mousavi 2008; De Ayala et al. 2005; Dozois et al. 1998), and severe (36 and above) based on total scores (De Ayala et al. 2005). For depression assessment, the Persian version of Beck Depression Inventory‐II (BDI‐II), which includes 21 statements, was used. This questionnaire has also been validated in Iran, with a Cronbach's alpha of 0.87 (Ghassemzadeh et al. 2005). Depression severity was categorized as minimal or mild (0–16), moderate (17–30), and severe (31 and above) based on the total score (Dozois et al. 1998).

### 2.3 Sleep Assessment

Individuals were asked about the number of hours they slept per night. According to the National Sleep Foundation (Foundation NS 2023), adequate sleep was considered as more than 7 h of sleep per day. Inadequate sleep was set as less than 7 h of sleep daily (Medicine AAoS 2015).

### 2.4 Dietary Assessment

Dietary intake was assessed by trained dietitians using a reliable and validated semi‐quantitative Food Frequency Questionnaire (FFQ) that included 65 food items (Ahmadnezhad et al. 2017). This questionnaire is designed to reflect the typical dietary intake of Iranians. Data were reported based on the frequency and amount of intake on a daily, weekly, monthly, rarely, and never basis. The analysis of macronutrient and micronutrient intake was conducted using Diet Plan 6 software (Forestfield Software Ltd., Horsham, West Sussex, UK). The validity and reproducibility of the FFQ applied in this study were evaluated previously in a subset of the MASHAD cohort. The results, including intra‐class correlation coefficients, Pearson's correlation coefficients, and a Cronbach's alpha of 0.67, demonstrated acceptable levels of reliability and reproducibility (Ahmadnezhad et al. 2017).

### 2.5 UPF Calculation

We assessed UPF consumption according to the NOVA classification. This system classifies foods based on their level of processing (Monteiro et al. 2016). Food and beverage items classified as UPF include non‐dairy drinks, industrial bread and cakes, fast food, salty snacks, potato chips, dairy beverages, processed meat, sauces, as well as sweets. Details are described in Table S1. To estimate each individual's daily UPF consumption (g/day), portion sizes and consumption frequencies were converted into grams using standard Iranian household measures, and the total amount of all UPF items was calculated.

### 2.6 Other Variables Assessments

Demographic factors (education, smoking, gender, age group, marital status), medical history (diabetes, dyslipidemia, hypertension), anthropometric data (body mass index), and physical activity levels (PAL) were assessed by healthcare professionals. To evaluate physical activity levels, a modified version of the Scottish Heart Health Study (SHHS)/MONICA questionnaire was employed. The relevant questions focused on the duration of activities during work, leisure time, and sleep. Additional details have been provided previously (Bahari et al. 2023). Confounding factors included hypertension (systolic blood pressure ≥ 140 mmHg, diastolic blood pressure ≥ 90 mmHg, or treated hypertension), diabetes (fasting plasma glucose ≥ 126 mg/dL or diagnosed type 2 diabetes), and dyslipidemia (low‐density lipoprotein cholesterol ≥ 130 mg/dL, triglycerides ≥ 150 mg/dL, high‐density lipoprotein cholesterol < 40 mg/dL for men, < 50 mg/dL for women, or treated dyslipidemia). All assessments were based on the methodology previously introduced by Asadi et al. (2020).

### 2.7 Statistical Analysis

Participants were categorized based on the quartiles cut‐off values of the UPF score. Performance of statistical analysis was done using SPSS‐25 software (SPSS Inc., IL, USA) and a *p*‐value of < 0.05 was established as statistically significant. The participants' characteristics across UPF score quartiles were displayed as means ± SDs for continuous variables. However, categorical variables were demonstrated as frequency numbers or percentages. To assess differences across UPF quartiles, one‐way analysis of variance (ANOVA) was used for continuous variables. As regards categorical variables, the chi‐square test was applied. Binary logistic regression was applied to estimate odds ratios (ORs) for sleep adequacy. Due to the ordinal nature of symptom severity levels and to allow estimation of odds across the full range of outcome categories, ordinal regression was used to evaluate the severity of anxiety and depression across UPF quartiles. A 95% confidence interval (CI) was considered for both crude and multivariable‐adjusted models. For the total population, crude represents the unadjusted model. Adjustments were made for the first model as for gender (male, female) as well as age (continuous). Model 2 received further adjustment regarding energy intake (continuous), marital status (married, single, widowed, or divorced), level of education (low, moderate, high), smoking status (current/ex−/non‐smoker), hypertension, and diabetes status (yes/no). The third model included all adjustments from Model 2, with additional adjustments specific to each outcome. Anxiety was additionally adjusted for depression and sleep adequacy; depression was further adjusted for anxiety and sleep adequacy. Sleep adequacy itself was also adjusted for factors of anxiety and depression. For sex‐stratified analyses, the same models were applied, except that gender was not included as an adjustment variable.

## 3 Results

The present study involved a total of 5476 participants (60% women). In this study, demographic and clinical features of study participants were compared across quartiles of UPF consumption (Table 1). The mean age, physical activity level, and sleep duration differed significantly across the UPF quartiles (*p* < 0.001). Individuals falling into the highest quartile (with the highest ultra‐processed food consumption) were younger and had lower levels of physical activity and adequate sleep in comparison with those in the other quartiles. Additionally, significant differences were found in the different groups of ultra‐processed food consumers regarding gender, education level, marital status, smoking status, blood pressure, and diabetes (all *p* < 0.001). No significant discrepancies were observed across the groups for depression score, anxiety score, or other variables (all *p* > 0.05).

**TABLE 1:** Baseline demographic and clinical features of study participants by UPF quartiles.

Variables	Ultra‐Processed Food				*p*
	Quartile1 (*n* = 1367)	Quartile2 (*n* = 1360)	Quartile3 (*n* = 1370)	Quartile4 (*n* = 1379)	
Age (year)	50.11 ± 8.47	48.39 ± 7.98	48.03 ± 7.88	47.52 ± 8.12	> 0.001
Body mass index (kg/m^2^)	27.86 ± 4.65	28.07 ± 4.74	27.94 ± 4.72	27.78 ± 4.68	0.44
Physical activity level	1.65 ± 0.29	1.58 ± 0.27	1.57 ± 0.27	1.55 ± 0.28	> 0.001
Nightly sleep (hours)	6.81 ± 1.52	6.98 ± 1.45	6.98 ± 1.47	6.88 ± 1.56	0.003
Nightly sleep					
Adequate	497 (36.36)	553 (40.66)	564 (41.17)	536 (38.87)	0.04
Inadequate	870 (63.64)	807 (59.34)	806 (58.83)	843 (61.13)	
Depression Score	12.70 ± 9.09	12.05 ± 9.15	12.44 ± 9.45	12.78 ± 9.61	0.17
Depression Score					
Mild or no depression	1118 (81.84)	1138 (83.86)	1121 (81.94)	1111 (80.68)	0.19
Moderate to severe	248 (18.16)	219 (16.14)	247 (18.06)	266 (19.32)	
Anxiety Score	10.79 ± 10.09	10.13 ± 9.38	10.25 ± 9.53	10.64 ± 9.87	0.24
Anxiety Score					
< 16	1032 (75.49)	1031 (75.98)	1061 (77.50)	1036 (75.24)	0.51
≥ 16	335 (24.51)	326 (24.02)	308 (22.50)	341 (24.76)	
Gender, *n* (%)					
Male	436 (31.89)	510 (37.50)	567 (41.39)	677 (49.09)	> 0.001
Female	931 (68.11)	850 (62.50)	803 (58.61)	702 (50.91)	
Marital status, *n* (%)					
Married	1238 (90.56)	1268 (93.24)	1282 (93.58)	1299 (94.20)	0.001
Single	8 (0.59)	5 (0.37)	1.(0.73)	1.(0.80)	
Widow	101 (7.39)	65 (4.78)	64 (4.67)	47 (3.41)	
Divorced	20 (1.46)	22 (1.62)	1.(1.02)	22 (1.60)	
Education level, *n* (%)					
Low	915 (66.93)	744 (54.74)	695 (50.77)	669 (48.58)	> 0.001
Moderate	363 (26.55)	453 (33.33)	509 (37.18)	532 (38.63)	
High	89 (6.51)	162 (11.92)	165 (12.05)	176 (12.78)	
Smoking status, *n* (%)					
None‐smoker	996 (72.86)	1009 (74.19)	909 (66.35)	808 (58.59)	> 0.001
Ex‐smoker	148 (10.83)	133 (9.78)	146 (10.66)	140 (10.15)	
Current smoker	223 (16.31)	218 (16.03)	315 (22.99)	431 (31.25)	
Hypertension, *n* (%)					
Yes	533 (39.13)	425 (31.34)	443 (32.43)	406 (29.44)	> 0.001
Diabetes, *n* (%)					
Yes	309 (22.92)	185 (13.74)	171 (12.66)	132 (9.66)	< 0.001
Dyslipidemia, *n* (%)					
Yes	1196 (87.94)	1175 (86.91)	1184 (87.06)	1180 (85.94)	0.49

*Note:* Continuous variables are presented as Mean ± SD, whereas categorical variables are depicted as counts (percentages). ANOVA was employed to examine continuous variables, and the chi‐square test was utilized for categorical variables.

Abbreviation: UPF, ultra‐processed food.

The differences in the consumption of UPF items across the quartiles are presented in Table 2. As UPF consumption increased, so did the intake of energy, as well as food and beverages, including non‐dairy beverages, cookies, dairy beverages, salty snacks, processed fast food, sauces, and sweets, all of which significantly increased across the quartiles (all *p* < 0.001).

**TABLE 2:** Primary ultra‐processed food items categorized by UPF quartiles.

Variables	Ultra‐processed food				*p*
	Quartile1 (*n* = 1367)	Quartile2 (*n* = 1360)	Quartile3 (*n* = 1370)	Quartile4 (*n* = 1379)	
Energy (kcal/day)	1736.54 ± 598.92	1949.23 ± 537.82	2060.73 ± 583.61	2291.31 ± 682.23	< 0.001
Non‐Dairy Beverages (gr/day)	6.35 ± 5.37	18.96 ± 9.92	53.78 ± 24.42	235.23 ± 252.68	< 0.001
Cookies (kcal/day)	6.35 ± 5.37	18.96 ± 9.92	53.78 ± 24.42	235.23 ± 252.68	< 0.001
Dairy Beverages (kcal/day)	2.89 ± 3.65	7.29 ± 8.83	10.60 ± 15.65	18.23 ± 44.05	< 0.001
Salty Snacks (kcal/day)	2.28 ± 3.38	4.85 ± 6.98	8.10 ± 17.04	13.23 ± 33.50	< 0.001
Processed Fast Food (kcal/day)	3.76 ± 6.45	11.70 ± 9.89	14.42 ± 16.63	20.61 ± 45.21	< 0.001
Sauces (kcal/day)	1.50 ± 2.86	2.51 ± 4.15	3.23 ± 5.65	4.00 ± 6.31	< 0.001
Sweets (kcal/day)	1.72 ± 3.39	4.13 ± 7.68	6.22 ± 13.65	7.95 ± 22.96	< 0.001

*Note:* Variables are presented as Mean ± SD. ANOVA was employed to compare groups.

Table 3 presents the results of ordinal regression, exploring the association between UPF intake and severity of depression across different quartiles of UPF. In the total population, no significant associations were found in the crude model (*p* < 0.05). However, after adjusting for confounding variables in the first model, participants in the higher quartile of UPF consumption had significantly higher odds of more severe depression, compared to the lowest quartile (OR = 1.23: 95% CI: 1.01–1.49). This association remained significant in Model 2 (OR = 1.25: 95% CI: 1.01–1.54) although it was no longer statistically significant in Model 3 (OR: 1.25, 95% CI: 0.99–1.57).

**TABLE 3:** Ordinal regression on the association between ultra‐processed food consumption and depression severity.

Variable			Ultra‐processed food				
			Q1	Q2	Q3	Q4	*p*‐trend
Depression	*Total population*						
	Crude	OR (95% CI)	1	0.87 (0.71–1.07)	0.99 (0.81–1.20)	1.08 (0.89–1.31)	0.26
		*p*	0.21	0.18	0.88	0.44	
	Model 1	OR (95% CI)	1	0.91 (0.74–1.12)	1.06 (0.87–1.29)	1.23 (1.01–1.49)	0.02
		*p*	0.03	0.37	0.59	0.04	
	Model 2	OR (95% CI)	1	0.97 (0.79–1.19)	1.10 (0.89–1.35)	1.25 (1.01–1.54)	0.02
		*p*	0.08	0.77	0.39	0.04	
	Model 3	OR (95% CI)	1	1.01 (0.80–1.26)	1.16 (0.92–1.46)	1.25 (0.99–1.57)	0.03
		*p*	0.17	0.97	0.21	0.07	
	*Males*						
	Crude	OR (95% CI)	1	1.01 (0.68–1.51)	0.98 (0.66–1.45)	1.07 (0.74–1.55)	0.73
		*p*	0.96	0.95	0.91	0.71	
	Model 1	OR (95% CI)	1	1.01 (0.68–1.50)	0.97 (0.66–1.43)	1.06 (0.73–1.54)	0.77
		*p*	0.96	0.98	0.88	0.75	
	Model 2	OR (95% CI)	1	1.01 (0.67–1.51)	0.93 (0.62–1.39)	1.00 (0.67–1.48)	0.91
		*p*	0.97	0.98	0.72	0.98	
	Model 3	OR (95% CI)	1	1.00 (0.65–1.55)	0.85 (0.55–1.31)	0.88 (0.58–1.35)	0.44
		*p*	0.81	0.99	0.45	0.56	
	*Females*						
	Crude	OR (95% CI)	1	0.86 (0.68–1.09)	1.08 (0.85–1.36)	1.30 (1.03–1.64)	0.01
		*p*	0.01	0.22	0.53	0.03	
	Model 1	OR (95% CI)	1	0.87 (0.69–1.11)	1.09 (0.87–1.38)	1.32 (1.04–1.67)	0.01
		*p*	0.01	0.26	0.46	0.02	
	Model 2	OR (95% CI)	1	0.94 (0.74–1.20)	1.16 (0.91–1.47)	1.38 (1.07–1.77)	0.01
		*p*	0.02	0.63	0.24	0.01	
	Model 3	OR (95% CI)	1	0.98 (0.75–1.28)	1.28 (0.98–1.68)	1.44 (1.09–1.90)	< 0.001
		*p*	0.01	0.86	0.07	0.01	

*Note:* For total population, crude: unadjusted; Model1: adjusted for gender, and age; Model2: Model1+ energy intake, marriage status, education level, smoking status, hypertension, and diabetes; Model3: Model2 + anxiety score, and sleep adequacy. For analyses stratified by sex, the same models were used, except that gender was not included as an adjustment variable. *p*‐value < 0.05 were considered as meaningful.

In males, no significant associations were found between UPF intake and depression severity in any of the analytical models (all *p* > 0.05). In females, the highest intake of UPF was significantly associated with increased odds of more severe depression compared to the lowest intake in all models. Specifically, in the fully adjusted model, females in the highest quartile of UPF consumption had 44% higher odds of more severe depression (OR = 1.44, 95% CI: 1.09–1.90).

The results of the study showed no significant association between ultra‐processed food consumption and severity of anxiety or sleep adequacy in the overall population, as well as in gender subgroups (men and women). In none of the models did the OR change significantly across quartiles of ultra‐processed foods (all *p* > 0.05) (Tables 4 and 5).

**TABLE 4:** Ordinal regression on the association between ultra‐processed food consumption and anxiety severity.

Variable			Ultra‐processed food				*p*‐trend
			Q1	Q2	Q3	Q4	
Anxiety	*Total population*						
	Crude	OR (95% CI)	1	0.98 (0.82–1.17)	0.91 (0.76–1.08)	1.01 (0.85–1.21)	0.92
		*p*	0.60	0.80	0.28	0.87	
	Model 1	OR (95% CI)	1	1.02 (0.86–1.23)	0.97 (0.81–1.17)	1.16 (0.97–1.39)	0.17
		*p*	0.22	0.80	0.76	0.10	
	Model 2	OR (95% CI)	1	1.09 (0.90–1.30)	1.00 (0.83–1.21)	1.18 (0.98–1.43)	0.17
		*p*	0.24	0.38	0.99	0.09	
	Model 3	OR (95% CI)	1	1.11 (0.91–1.37)	0.97 (0.78–1.19)	1.07 (0.86–1.33)	0.87
		*p*	0.51	0.30	0.74	0.54	
	*Males*						
	Crude	OR (95% CI)	1	0.91 (0.63–1.32)	1.07 (0.75–1.52)	1.28 (0.92–1.78)	0.61
		*p*	0.18	0.63	0.71	0.15	
	Model 1	OR (95% CI)	1	0.90 (0.63–1.30)	1.05 (0.74–1.50)	1.26 (0.90–1.75)	0.08
		*p*	0.20	0.58	0.77	0.18	
	Model 2	OR (95% CI)	1	0.92 (0.63–1.33)	1.04 (0.73–1.49)	1.23 (0.87–1.75)	0.14
		*p*	0.34	0.64	0.84	0.24	
	Model 3	OR (95% CI)	1	0.86 (0.57–1.30)	1.00 (0.68–1.49)	1.21 (0.82–1.77)	0.19
		*p*	0.34	0.48	1.00	0.34	
	*Females*						
	Crude	OR (95% CI)	1	1.07 (0.87–1.31)	0.93 (0.75–1.15)	1.10 (0.88–1.36)	0.72
		*p*	0.48	0.54	0.51	0.41	
	Model 1	OR (95% CI)	1	1.08 (0.88–1.32)	0.94 (0.76–1.17)	1.11 (0.90–1.38)	0.63
		*p*	0.46	0.49	0.59	0.34	
	Model 2	OR (95% CI)	1	1.16 (0.94–1.44)	0.99 (0.79–1.24)	1.15 (0.91–1.45)	0.51
		*p*	0.31	0.16	0.93	0.24	
	Model 3	OR (95% CI)	1	1.24 (0.98–1.57)	0.96 (0.75–1.23)	1.00 (0.77–1.30)	0.56
		*p*	0.14	0.08	0.74	0.99	

*Note:* For total population, crude: unadjusted; Model1: adjusted for gender, and age; Model2: Model1+ energy intake, marriage status, education level, smoking status, hypertension, and diabetes; Model3: Model2 + depression score, and sleep adequacy. For analyses stratified by sex, the same models were used, except that gender was not included as an adjustment variable. *p*‐value < 0.05 were considered as meaningful.

**TABLE 5:** Binary logistic regression on the association between ultra‐processed food consumption and sleep adequacy.

Variable			Ultra‐processed food				*p*‐trend
			Q1	Q2	Q3	Q4	
Sleep adequacy	*Total population*						
	Crude	OR (95% CI)	1	0.82 (0.71–0.96)	0.82 (0.70–0.96)	0.90 (0.77–1.05)	0.18
		*p*	0.04	0.02	0.01	0.16	
	Model 1	OR (95% CI)	1	0.83 (0.71–0.97)	0.82 (0.71–0.96)	0.89 (0.76–1.05)	0.19
		*p*	0.06	0.02	0.02	0.17	
	Model 2	OR (95% CI)	1	0.81 (0.69–0.95)	0.81 (0.69–0.96)	0.91 (0.77–1.08)	0.31
		*p*	0.03	0.01	0.01	0.27	
	Model 3	OR (95% CI)	1	0.81 (0.69–0.96)	0.81 (0.69–0.95)	0.90 (0.76–1.06)	0.23
		*p*	0.03	0.01	0.01	0.20	
	*Males*						
	Crude	OR (95% CI)	1	0.89 (0.68–1.16)	0.82 (0.63–1.07)	0.95 (0.73–1.22)	0.69
		*p*	0.48	0.38	0.15	0.66	
	Model 1	OR (95% CI)	1	0.90 (0.69–1.18)	0.84 (0.65–1.09)	0.97 (0.75–1.25)	0.83
		*p*	0.53	0.44	0.19	0.80	
	Model 2	OR (95% CI)	1	0.86 (0.66–1.13)	0.80 (0.61–1.04)	0.94 (0.72–1.23)	0.68
		*p*	0.34	0.29	0.10	0.64	
	Model 3	OR (95% CI)	1	0.86 (0.66–1.14)	0.79 (0.60–1.04)	0.93 (0.71–1.22)	0.61
		*p*	0.33	0.29	0.09	0.59	
	*Females*						
	Crude	OR (95% CI)	1	0.78 (0.65–0.95)	0.80 (0.66–0.97)	0.82 (0.67–1.00)	0.05
		*p*	0.43	0.01	0.02	0.05	
	Model 1	OR (95% CI)	1	0.80 (0.66–0.97)	0.83 (0.68–1.00)	0.85 (0.70–1.05)	0.13
		*p*	0.11	0.03	0.05	0.13	
	Model 2	OR (95% CI)	1	0.80 (0.66–0.98)	0.84 (0.69–1.03)	0.90 (0.73–1.12)	0.38
		*p*	0.14	0.03	0.09	0.35	
	Model 3	OR (95% CI)	1	0.80 (0.66–0.98)	0.84 (0.68–1.02)	0.89 (0.71–1.10)	0.30
		*p*	0.14	0.03	0.08	0.27	

*Note:* For total population, crude: unadjusted; Model1: adjusted for gender, and age; Model2: Model1+ energy intake, marriage status, education level, smoking status, hypertension, and diabetes; Model3: Model2 + depression score, and anxiety score. For analyses stratified by sex, the same models were used, except that gender was not included as an adjustment variable. *p*‐value < 0.05 were considered as meaningful.

## 4 Discussion

In this cross‐sectional study, no statistically significant association was observed between UPF consumption and depression severity in the total population. However, females in the highest category of UPF intake had significantly higher odds of more severe depression, compared to the lowest category. UPF consumption was not significantly associated with anxiety severity and sleep adequacy in the total population, males, or females.

The results of the present study are aligned with some previous research which examined the link between UPF consumption and depression. For example, Mengist et al. found a positive association between UPF consumption and depressive symptoms (Mengist et al. 2025). Similarly, Ghernati et al. observed associations with both depression and anxiety (Ghernati et al. 2025). Additionally, in earlier cohort studies, including Adjibade et al. a 10% increase in the intake of ultra‐processed foods was found to be related to a 21% increase in the risk of developing depression symptoms over a period of 5 years of follow‐up (Adjibade et al. 2019). Likewise, in a study by Gómez et al. participants who consumed higher amounts of UPF showed a 33% higher risk of developing depression in a 10‐year period (Gómez‐Donoso et al. 2020). These findings are further supported by an umbrella review by Lane et al. which highlighted the relationship between UPFs and depression (Lane et al. 2024), and a meta‐analysis by Barbaresko et al. that observed a negative link between UPF intake and mental health (Barbaresko et al. 2024). However, some other studies have reported conflicting results. For instance, findings from a Korean study reported no significant association between UPF consumption and depressive symptoms in men (Lee and Choi 2023). Similarly, research conducted in the United States found no association among individuals with higher physical activity levels (Zheng et al. 2020). These discrepancies could have resulted from variations in the design of the study, and contextual factors such as age, sex, and culture might have played moderating roles.

The finding that a significant relationship was observed between UPF consumption and depression only in females may be partly explained by previous research working on the role of gender differences in how diet impacts mental health (Lee and Choi 2023). Previous studies indicated that women are generally prone to experience depression and might be more sensitive to dietary factors due to hormonal and psychosocial influences (Wardle et al. 2004; Zheng et al. 2020). Additionally, women may be more likely to report depressive symptoms, which could explain why the link between UPFs and depression severity is stronger in females (Lee and Choi 2023; Warren 2024).

The relationship between UPF consumption and depression has been proposed to involve inflammatory processes and metabolic disturbances, as suggested by previous studies. UPFs are associated with chronic nutrition‐related diseases such as obesity and metabolic syndrome, both of which are bidirectionally linked to depression (Milaneschi et al. 2019). Proposed biological mechanisms include hyperactivity of the HPA axis, excessive cortisol secretion, immune‐inflammatory signaling triggered by pro‐inflammatory diets, leptin and insulin resistance, and interference with the gut‐brain microbiome axis (Foster and Neufeld 2013; Sánchez‐Villegas et al. 2015; Sanchez‐Villegas et al. 2018). UPF consumption also results in a reduced intake of vital nutrients such as magnesium, potassium, vitamins, and fiber. Deficiencies in these nutrients intensify the risk of depression (Li et al. 2018; Xu et al. 2018). In addition, foodborne contaminants generated during food processing and phthalates present in packaging have been reported to be linked to depression symptoms (Canella et al. 2018). High‐level intake of refined grains, simple sugars, and saturated and trans fats contributes to mental disorders by inducing systemic inflammation, oxidative stress, and mitochondrial dysfunction (DiNicolantonio et al. 2018; Duan et al. 2018). A decrease in fiber intake can also disrupt the gut microbiome balance through the gut‐brain microbiome axis, which can further boost the risk of depression (Ghaisas et al. 2016; Koopman and El Aidy 2017). However, it is important to note that these mechanisms are presented to contextualize the current findings within the broader scientific literature.

Moreover, limited research is available on the relationship between UPF consumption and the risk of developing anxiety (Mazloomi et al. 2023; Lane et al. 2022). While one study showed a positive association between UPF consumption and anxiety (Lane et al. 2022), another study did not confirm such a link (Mazloomi et al. 2023). This discrepancy may be because of the limitations of cross‐sectional designs and smaller sample sizes in the studies, which do not effectively capture causal relationships. Furthermore, energy intake plays an important role. Some evidence indicates that the amount of UPFs consumed may be the main factor influencing mental health. This suggests that high levels of UPF consumption, often associated with excessive energy intake, could contribute to the link between UPFs and mental disorders (Cediel et al. 2021; Neri et al. 2022).

We observed no significant association between sleep adequacy and UPF consumption in adults in this study. While some previous studies have reported a positive association between high UPF consumption and a rising risk of insomnia (Lane et al. 2022; Duquenne et al. 2024), some studies have shown that this association is significant in adolescents but not in adults (Pourmotabbed et al. 2024). This variation may be due to differences in dietary patterns, greater metabolic sensitivity in adolescents, and physiological adaptations in adults (Andreeva et al. 2023). Adolescents are more prone to blood sugar fluctuations and their negative effects on sleep due to their higher consumption of added sugars and high‐calorie foods, whereas in adults, better regulation of circadian rhythms and more stable dietary habits may reduce these effects (Barreto et al. 2024). Based on previous studies, several mechanisms have been proposed through which high UPF consumption may be negatively related to sleep quality. These include reduced tryptophan and melatonin intake (Bae et al. 2016; Losso et al. 2018), increased glycemic index and blood sugar fluctuations (Seaquist et al. 2013), disruption of gut microbiome composition and neurohormonal pathways (Wang et al. 2024), as well as exposure to endocrine disrupting compounds (e.g., bisphenol A) (Beydoun et al. 2016). Also, the consumption of saturated fats and added sugars in UPF may reduce slow‐wave sleep (SWS) and increase nocturnal awakenings (St‐Onge et al. 2016).

Our findings are also consistent with some recent studies conducted in Iran. Hosseininasab et al. reported that higher UPF consumption was linked to a decreased quality of life among Iranian women (Hosseininasab et al. 2024). Hajmir et al. showed an interaction between UPF intake and genetic risk factors influencing mental health and sleep quality (Hajmir et al. 2022). Additionally, other studies highlighted the role of unhealthy dietary patterns, including high UPF consumption, in increasing the depression and anxiety risk in Iranian adults, underscoring the need for culturally tailored interventions (Bazyar et al. 2020; Janmohammadi et al. 2021; Bakhtiyari et al. 2013; Saeidlou et al. 2021). Strengths of the present study include its relatively large sample size, which allowed for stronger statistical analyses and increased the generalizability of the findings. The use of the standardized NOVA classification system for assessing food processing levels is also one of the positive points of this study, as it improved the accuracy of food consumption measurement and facilitated comparisons with the results of other studies. Additionally, efforts to control for confounding variables, such as demographic and lifestyle factors, helped enhance the accuracy of the findings.

However, the present study was also not without limitations. The cross‐sectional design of the study restricted allowance for definitive identification of a causal relationship between UPF consumption and depression, raising the possibility of reverse causation. Another drawback of this study is that data on the relationship between food intake and the severity of depression experienced by the participants were self‐reported, which may have introduced potential recall bias and misreporting. The FFQ tool used was also not specifically designed to assess the consumption of ultra‐processed foods, which could reduce the accuracy of the values associated with UPF intake. Additionally, the presence of other possible confounding factors, including medication use, psychiatric history in individuals and their relatives, socioeconomic status, psychological inclinations or personality traits, genetic predispositions, and biological factors are likely to have affected the results. Moreover, sleep adequacy was merely evaluated based on sleep duration because information on sleep quality, sleep disorders, and the use of sleep medications was not collected, which may limit the comprehensive assessment of sleep outcomes. Regarding UPF consumption, a number of participants lacked information on chocolate intake, an essential component in calculating the UPF index. These participants were excluded from the analysis, and no imputation methods were applied.

## 5 Conclusion

All in all, the final findings of our investigation suggest no statistically significant association between UPF consumption and depression severity in the general population. However, women taking the highest amount of UPF presented significantly greater chances of experiencing depression more severely than those with the lowest intake. No significant associations were found between UPF intake and either anxiety or sleep adequacy. While further research, particularly randomized controlled trials, is needed, it can be concluded that a diet rich in UPF represents an undesirable mix of biologically active food additives and a low content of essential nutrients, which can have negative impacts on mental health, especially in women.

## Author Contributions

**Niloufar Abdollahpour:** visualization (lead), writing – original draft (lead). **Seyedeh Atieh Mousavi Fard:** investigation (equal). **Alae Salahmanesh:** investigation (equal). **Hossein Hatamzadeh:** investigation (equal). **Reza Moeini:** investigation (equal). **Sara Saffar Soflaei:** conceptualization (equal), data curation (equal). **Najmeh Seifi:** conceptualization (equal), formal analysis (lead), methodology (lead), validation (equal), writing – review and editing (equal). **Majid Ghayour‐Mobarhan:** conceptualization (equal), funding acquisition (lead), project administration (lead), supervision (lead), writing – review and editing (equal).

## Ethics Statement

All experiments were performed in accordance with the declaration of Helsinki and Mashhad University of Medical Sciences ethical guidelines and regulations. The research protocol was approved by the School of Medicine, Mashhad University of Medical Sciences, Biomedical Research Ethics Committee (IR.MUMS.MEDICAL.REC.1403.412).

## Consent

All participants signed a written informed consent before participating in the study.

## Conflicts of Interest

The authors declare no conflicts of interest.

## Supporting information


Data S1.

**Table S1:** Food and beverage product items included as ultra‐processed food in the present study.

## Data Availability

The datasets generated and/or analyzed during the current study are not publicly available due to university data ownership policies, but are available from the corresponding author on reasonable request.
