# Supplementary material for: The Association between Ultra‐Processed Foods and Depression, Anxiety and Sleep in Adults: A Cross‐Sectional Study in Iran
Source: Food Sci Nutr. 2025 Jul 20;13(7):e70316. doi: 10.1002/fsn3.70316 (PMC12277236; doi:10.1002/fsn3.70316)
Supplement: Supplementary file 1 — Data S1. [file FSN3-13-e70316-s002.pptx]

## Slide 1
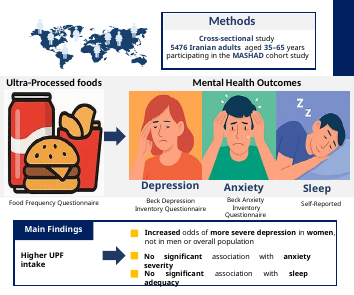

Methods
Cross-sectional study
5476 Iranian adults aged 35–65 years participating in the MASHAD cohort study
Ultra-Processed foods
Mental Health Outcomes
Depression
Anxiety
Sleep
Beck Anxiety
 Inventory Questionnaire
Beck Depression Inventory Questionnaire
Food Frequency Questionnaire
Self-Reported
Main Findings
Increased odds of more severe depression in women, not in men or overall population
Higher UPF intake
No significant association with anxiety severity
No significant association with sleep adequacy
