# Supplementary material for: The Association between Ultra‐Processed Foods and Depression, Anxiety and Sleep in Adults: A Cross‐Sectional Study in Iran
Source: Food Sci Nutr. 2025 Jul 20;13(7):e70316. doi: 10.1002/fsn3.70316 (PMC12277236; doi:10.1002/fsn3.70316)
Supplement: Supplementary file 2 — Table S1: Food and beverage product items included as ultra‐processed food in the present study. [file FSN3-13-e70316-s001.docx]

*Supplementary Table 1. Food and beverage product items included as ultra-processed food in the present study*

| Food groups | Food and beverage items |
| --- | --- |
| Non-dairy Beverages | Soda + Non-alcoholic malt drink + Diet soda + Instant coffee |
| Industrial bread and cakes | Cookie + Cake |
| Dairy beverages | Ice cream + Milk coffee |
| Potato chips and salty snacks | Potato chips + Cheese puffs |
| Processed meat and fast food | Pizza + Processed meat products |
| Sauces | Salad dressing |
| Sweets | Chocolate |

Definition of ultra-processed food items adapted from the NOVA classification retrieved from: Moubarac, Jean-Claude, et al. "Food classification systems based on food processing: significance and implications for policies and actions: a systematic literature review and assessment." Current obesity reports 3.2 (2014): 256-272.
